# Advances in Structural Reliability Analysis of Solid Propellant Grain: A Comprehensive Review

**DOI:** 10.3390/polym17152039

**Published:** 2025-07-26

**Authors:** Chenghu Tang, Hongfu Qiang, Tingjing Geng, Xueren Wang, Feng Zhang

**Affiliations:** 1 Zhijian Laboratory, Rocket Force University of Engineering, Xi’an 710025, China; chenghutang@xaau.edu.cn (C.T.); qiang@263.net (H.Q.); gengtingjing2022@163.com (T.G.); 2 School of Aerocraft, Xihang University, Xi’an 710077, China; 3 School of Mechanics and Transportation Engineering, Northwestern Polytechnical University, Xi’an 710129, China; nwpuwindy@nwpu.edu.cn

**Keywords:** solid rocket motor, solid propellant grain, failure mode analysis, failure analysis, reliability analysis

## Abstract

Solid propellant grain, as a typical polymer, are the thrust generation devices and core load-bearing components of solid rocket motor (SRM) and are also known as SRM grain. They are constantly exposed to extreme service environments such as high temperatures, high pressures, and dynamic shocks, and have a relatively high failure rate in the field use of SRM. Its life and reliability are the shortcomings that restrict the improvement of weapons and equipment capability in China at present. This paper summarizes the typical fault types of SRM grain at present, and compares and analyzes the research progress of reliability design and analysis technology, reliability optimization technology, life test technology and reliability evaluation technology of SRM grain at home and abroad; This paper analyzes the deficiencies and reasons in the research and application of SRM grain reliability technology in China, and points out the technical difficulties and challenges faced by the integrated design of performance and reliability of SRM independent innovation design according to the needs of the forward research and development system of SRM. Based on the existing design level and industrial foundation in China, the basic research suggestions that should be carried out to consolidate the design ability of SRM grain in China are given.

## 1. Introduction

As an important part of solid rocket motor (SRM), solid propellant grain structure is mainly responsible for providing the required thrust for SRM and ensuring the interior ballistic characteristics of SRM, so as to generate enough power to ensure the stable operation of rocket device and successfully complete the launching mission. SRM is mainly composed of engine combustion chamber shell, heat insulation layer, grain, nozzle and ignition device. The grain structure is mainly composed of combustion agent, oxidant and other components, which are usually high molecular polymer materials and have viscoelastic properties [1]. According to statistics, among all kinds of faults of SRM, the faults of grain structure account for a relatively high proportion. Therefore, how to further improve the quality, reliability and safety of grain structure, strengthen the research and development of reliability technology of weapons and equipment, and form the comprehensive design ability of high reliability and long-life solid propellant grain will be an important goal of the new generation SRM grain structure design [2].

In recent years, great technological breakthroughs and important research achievements have been made in SRM grain in Europe and America, which is mainly due to the detailed reliability design in every stage of product development from scheme demonstration to design, including: (1) In the stage of scheme demonstration of SRM, the product reliability requirements have been formulated in detail, and the repair rate, durability test time and accelerated life test time have been defined in detail. (2) Failure Mode, Effects and Criticality Analysis (FMECA) and Fault Tree Analysis (FTA) are carried out in the project development stage. (3) A full reliability test plan is made, including accelerated cycle durability test and vibration test. A large number of in-depth reliability technology research work in product development in European and American aerospace power countries have formed a perfect reliability research and development system, thus greatly improving the reliability of SRM [3]. In the early days, domestic SRM were mainly designed in reverse direction, but no forward reliability design system was formed. The reliability technology of SRM grain was far behind that of foreign countries, and the technical index of product life during the first turn-over period was far behind the advanced level of foreign countries. Because there is no positive reliability design system in the development stage of SRM grain in China, passive improvement measures after product failure are still adopted, such as exciting failure in test-improved design, failure in use-improved design, etc., that is, passive improvement afterwards, and sufficient reliability technology is lacking to expose and eliminate faults in the design stage. In the future, the severe launching conditions and harsh flight environment of the new generation of SRM will further aggravate the failure of SRM grain [4].

At present, the design focus of SRM grain in China has changed from high-performance independent design to high-reliability and long-life design [5]. Reliability design, as a systematic project, has long lacked attention to reliability design in China, and has not established a perfect and effective reliability database of SRM grain. The only remaining data still have many subjective and objective uncertainty problems, such as unclear data, incomplete data, wrong records, etc., so it is impossible to effectively apply abundant test data resources in research and service to design, thus failing to form an effective iteration of product reliability design [6]. In addition, the performance, life and reliability parameters in product design have not been designed cooperatively, and there is no standardized and feasible reliability design criterion in each stage. Only the reliability index (mainly life) of products is preliminarily verified in the test and acceptance of products, ignoring the core idea that reliability must run through the whole design process of products. In the future, the working environment of SRM will become more and more severe, and the grain will face more failure modes, more complex failure mechanisms and more uncertain coupling factors, which need to be solved urgently [7].

This paper introduces the structural characteristics, fault analysis and current status of reliability development of SRM propellant grain. It specifically analyzes the domestic and international research results related to the reliability of the propellant grain. Firstly, it briefly introduces the structural features of the propellant grain and conducts fault analysis on the common failure modes that occur during the design, manufacturing and storage of the grain. Secondly, it reviews the reliability issues of the propellant grain throughout its entire service life, including aspects such as reliability design, reliability optimization, reliability testing and reliability assessment. Finally, it summarizes and looks forward to the existing problems, main technical challenges, and future research directions in the reliability analysis of solid propellant grain.

## 2. Structure and Fault Analysis of Grain of SRM

### 2.1. Brief Introduction of SRM Grain

**Figure 1:**
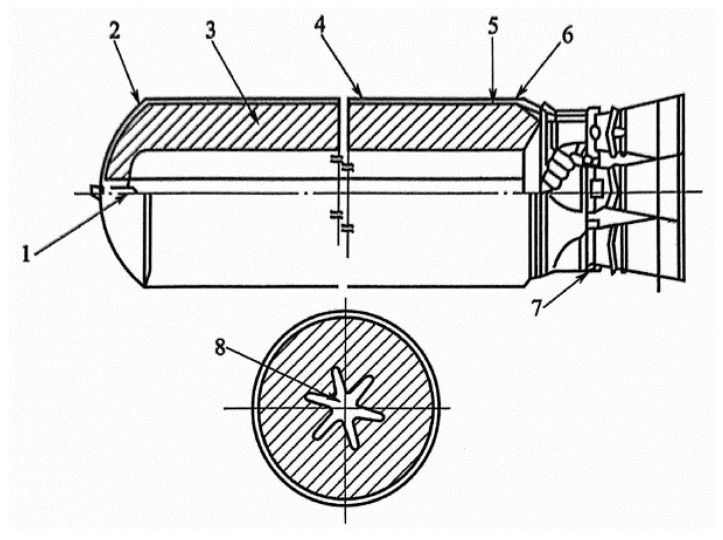
Typical SRM structure; 1—igniter; 2—artificial debonding layer; 3—grain; 4—metal shell; 5—internal insulation; 6—external insulation; 7—nozzle; 8—charge passage.

The SRM system is mainly composed of grain, combustion chamber shell (including metal and fiber reinforced composite shell), nozzle, ignition device, installation accessories, etc. [8]. According to the requirements, some engines also include thrust direction control and safety ignition mechanism. For multi-stage engines, connecting skirts are also required, as shown in Figure 1.

**Figure 2:**
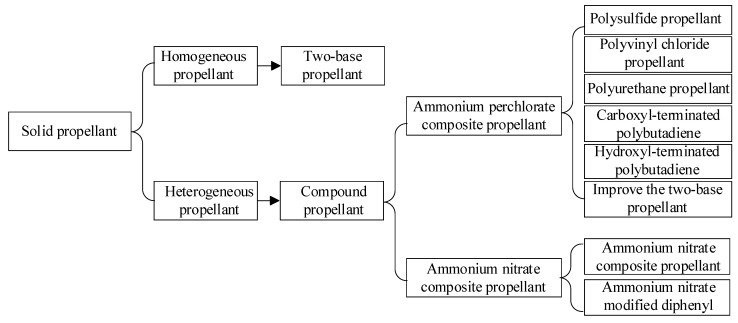
Classification of solid propellants.

As the power source of SRM, grain is the key structure of SRM and plays a vital role in the development of solid rocket [9]. The grain contains oxidant and combustion agent, which releases a large amount of hot gas through regular combustion, thus forming an emitting solid dense material. Grain is a solid propellant with a certain geometric shape and size, also known as solid propellant grain. According to whether there is a phase interface between the components of solid propellant, it can be divided into two categories: homogeneous propellant and heterogeneous propulsion [10], as shown in Figure 2.

Double-base propellants have the general properties of solid propellants, i.e., High energy, density of 1540~1650 kg/m^3^ and actual specific impulse of 1666~2156 N·s/kg. It has good combustion performance, and the combustion speed and pressure index can be close to zero; It has good mechanical properties, interior ballistic properties, technological properties and good stability; Its outstanding advantages are uniform texture, uniform structure and good reproducibility, which can meet the needs of tactical rockets and missiles [11,12].

**Figure 3:**
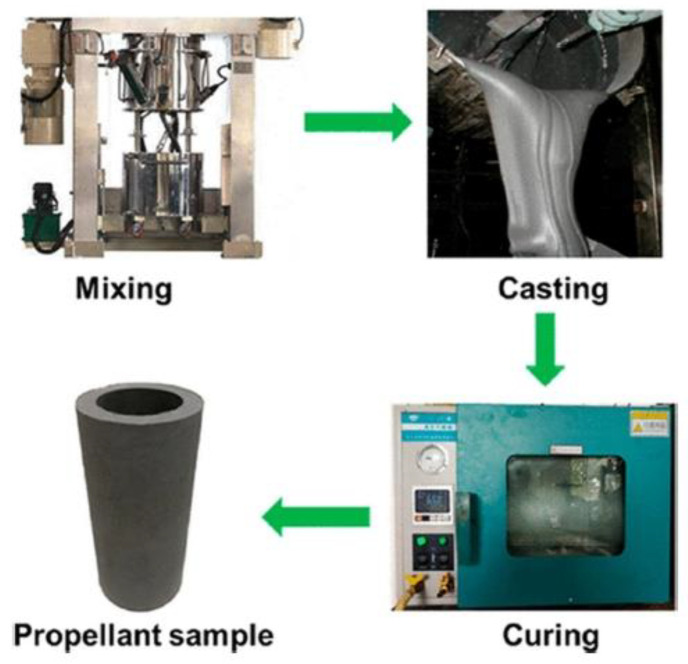
Manufacturing process of HTPB propellant.

Composite solid propellant is based on high polymer, mixed with oxidizer and metal fuel. Its actual specific impulse can reach 2450~2500 N·s/kg, its density is 1800 kg/m^3^, and it has good mechanical properties. According to the type of binder polymer, it can be divided into PVC, polyurethane, polybutadiene, and other propellants. It is a polymer composite filled with solid particles, which is usually obtained by crosslinking and curing liquid prepolymer [13], as shown in Figure 3. It can be made into a large SRM with shell bonding by pouring process, and is widely used in strategic missiles, tactical missiles, rockets and space vehicles.

**Figure 4:**
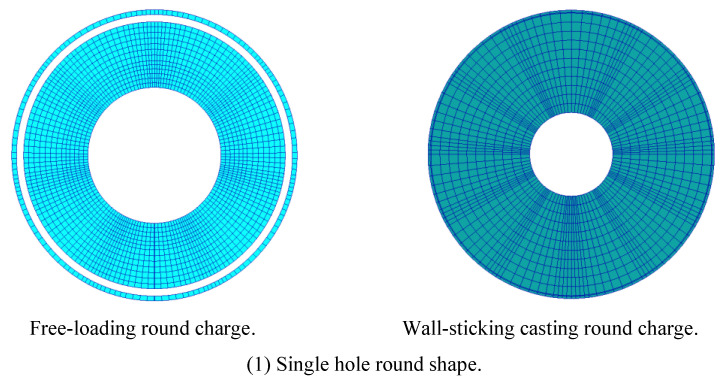

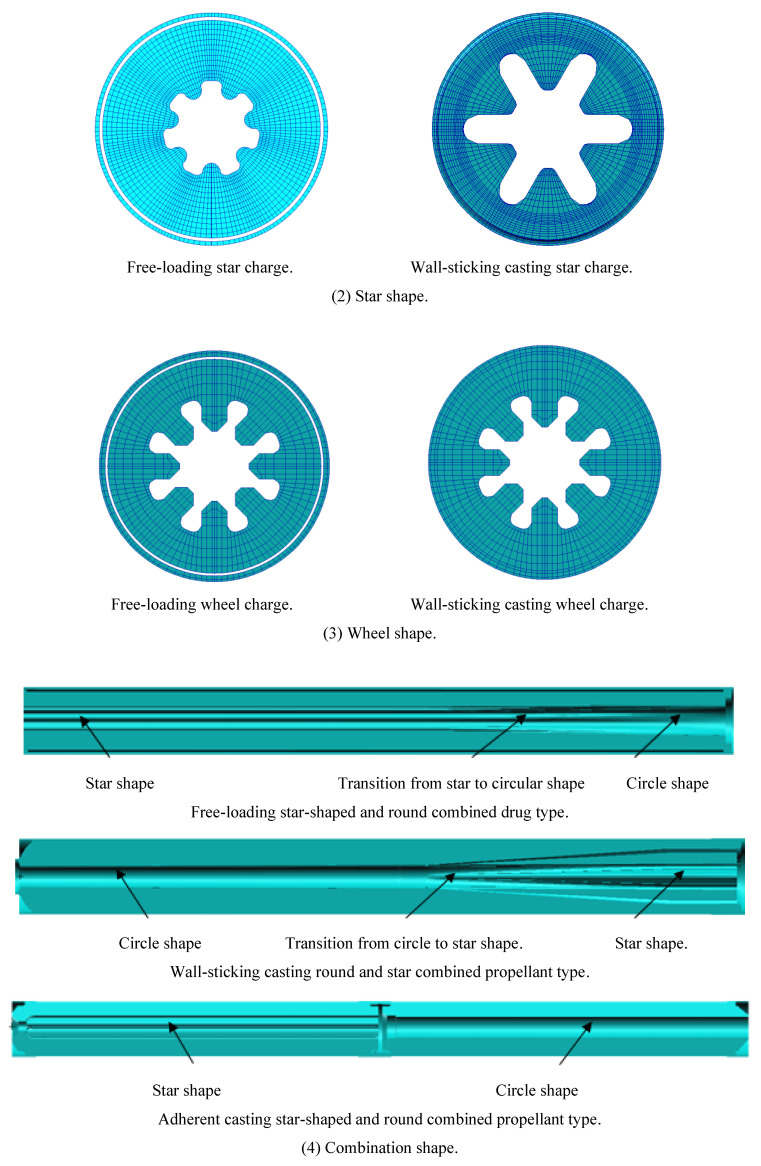
Typical charge types.

According to the law of burning area changing with time, the grain can be divided into constant surface, increasing surface and decreasing surface burning grain; According to the position of burning surface, it is divided into end face, side face and end-side burning grain; According to the projection number of burning surface normal in space rectangular coordinate system, it can be divided into one-dimensional, two-dimensional and three-dimensional grain; According to the shape of grain, it can be divided into cylindrical and spherical grain. Typical grain types include single-hole round, star-shaped, wheel-shaped and combined grain types [14], as shown in Figure 4.

The primary objective of simplified models for solid propellant combustion is to capture the general trends of burning rate variation with pressure and initial temperature. These simplified models are particularly valuable for addressing multidimensional transient problems. To achieve this, specific approximations are employed to derive analytical expressions for pressure as a function of burning rate. Within the condensed phase, under the assumptions of constant thermophysical properties and steady-state conditions, the mass and energy conservation equations, respectively, reduce to [15]:

(1) $${\dot{m}}^{\prime \prime }=\rho_{c}r_{b}$$

(2) $$\lambda_{c}\frac{d^{2}T}{dx^{2}}-\rho_{c}r_{b}c_{c}\frac{dT}{dx}+Q_{c}{\dot{w}}_{c}(x)=0$$

subject to the boundary conditions: $T=T_{0}$ at x=−∞, $T=T_{s}$ at x=0, where $\lambda_{c}$ is the thermal conductivity, $c_{c}$ the specific heat, and $Q_{c}$ the heat of reaction per unit mass in the condensed phase.

The working principle of the grain is that the SRM is composed of the ignition process of the charge, the combustion process and the flow process of the gas in the nozzle. When the grain burns in the combustion chamber, its chemical energy is converted into heat energy to generate high temperature and high pressure gas; The gas expands and accelerates through the nozzle, which converts the heat energy into kinetic energy, and the gas ejected backward at high speed gives the engine a reaction force, thus completing the propulsion process of rockets and missiles [16].

### 2.2. Typical Fault Analysis of SRM Grain

SRM will experience severe launching conditions and a complex flight environment during its working process. As a key power source, the failure problem of grain structure will be very prominent [17]. Up to now, a positive reliability design system has not been formed in grain structure design, which leads to the fact that reliability design theory has not been applied to the whole process of product development in grain design, which will lead to high failure frequency of the developed grain structure [18]. In this paper, the typical failure modes of SRM grain are summarized as follows.

#### 2.2.1. Cracks Appear in Grain

**Figure 5:**
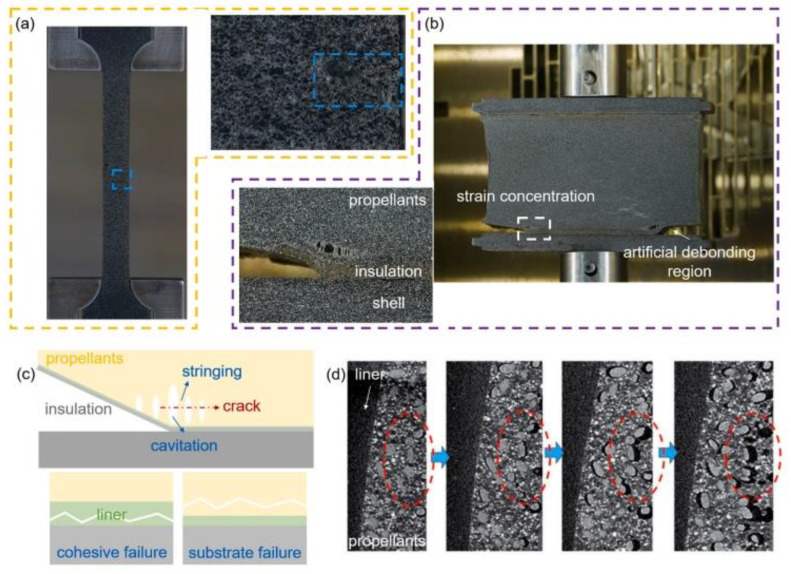
The crack propagation observed by the in situ digital image. (**a**) Failure of the solid propellants. (**b**) Debonding of the propellant-liner interface. The liner is not marked in the figure due to its tiny thickness compared with the propellants and the insulation. (**c**) A diagrammatic sketch of the substrate failure of the propellant-liner interface and the difference between the cohesive failure and the substrate failure. (**d**) The μ-CT observation of the substrate failure of the propellant liner interface [19].

According to the research on defects in SRM, it is agreed that cracks are the main factor leading to abnormal performance of solid propellant grain defects. The crack situation is shown in Figure 5 [19]. The crack in the grain of SRM provides extra combustion area for the combustion end face, and the combustion inside the crack will be affected by the pressure in the crack, erosive combustion and other factors, which makes the combustion process significantly different from the normal combustion in the grain channel, which leads to the abnormal local flow field in the engine, makes the internal ballistic parameters of the engine deviate from the design value, and finally affects the external ballistic parameters of the solid rocket [20]. In addition, the combustion on the crack surface will lead to the unstable propagation of the crack, and detonation will occur in severe cases.

During the launch and flight of solid rocket, after igniting the SRM, the pressure in the combustion chamber increases sharply, thus forming the effects of high pressure, large impact and strong vibration in the inner channel of the grain [21]. These interactions make the grain begin to form cracks, and the continuous action makes the cracks further expand until the grain finally breaks. In the study of grain fracture, the fracture problem under the dynamic effect including inertia and strain rate effect is mainly considered. One is the crack dynamic initiation problem, that is, the crack is stable and the external force changes rapidly with time, such as vibration, impact, wave and so on; The other is propagation crack problem or moving crack problem, that is, the external force is constant and the crack propagates rapidly, such as acceleration of crack propagation, crack arrest and bifurcation, etc. In the study of these two kinds of problems, it is necessary to consider the inertia effect in their motion equations.

**Figure 6:**
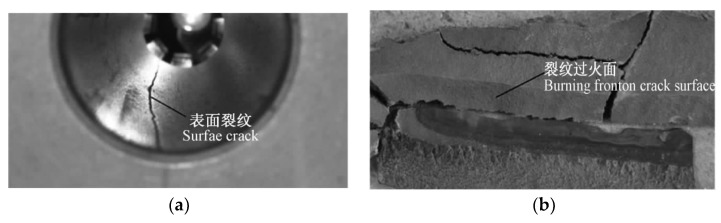
Schematic diagram of ignition test for solid propellant grain with cracks. (**a**) Cracks on the surface of the crystal grains. (**b**) Diagram showing the cracks after grain dissection [23].

At present, some theories have been put forward to analyze the crack of SRM grain. By analyzing the force of solid propellant, a model is established to study the relationship between stress, strain, displacement and material characteristic parameters or the damage of material when solid propellant is subjected to impact load or static pressure. Liu and Li studied the influence of grain crack on the working process of SRM, as shown in Figure 6 [22,23]. However, these studies can not quantitatively predict how cracks form, how to propagate, how to transform to large deformation or fragmentation, and have not given a consensus formula for calculating crack stress, nor has it quantitatively given how large cracks will bring danger to the use of grain, and there are few studies on the mutual coupling between multiple cracks. The research in this field cannot effectively guide the grain production technology, which needs further in-depth study.

Not only can the ignition impact bring damage to the grain, but also the casting grain has great danger in the process of curing and cooling, which may cause tiny cracks in the production of the grain and bring great hidden dangers to the normal use of SRM. In order to minimize the stress concentration in curing and cooling of cast rocket engine, the stress relief cover is often added to solve such problems. After the application of stress relief cover, there are few studies on how the stress in each part changes and how the stress relief is affected. To solve this problem, Sun carried out numerical simulation research on crack of SRM grain, which laid a foundation for studying crack propagation law, and studied the stress–strain field during solidification and cooling of grain [20].

#### 2.2.2. Debonding Occurs at the Grain Interface

**Figure 7:**
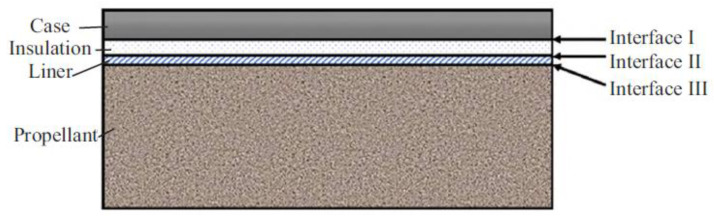
Interface structure of SRM.

The structure of SRM is formed by bonding and pouring four parts: shell, thermal insulation, liner and solid propellant from outside to inside. As shown in Figure 7, the interface between shell and thermal insulation is called industrial interface, the interface between thermal insulation and liner is called interface II, and the interface between liner and propellant is called interface III. During the long-term storage process, the interface adhesion performance of the engine will decline due to stress concentration and component migration, and gradually develop into defects such as cracks or voids, which will lead to interface failure and affect the overall performance and storage life of the engine [24]. Interfacial storage performance is an important part of engine storage performance research, and it is also a difficult point in engine storage performance research. There are three bonding interfaces of propellant-liner, liner-insulation and insulation-engine shell for wall-casting engine. During storage, the aging of propellant, the migration of components at the interface between propellant and liner, the change in environmental conditions and other factors will lead to the interface debonding of grain structure, and the engine with debonding defects is prone to fire and even explosion accidents during ignition [25].

**Figure 8:**
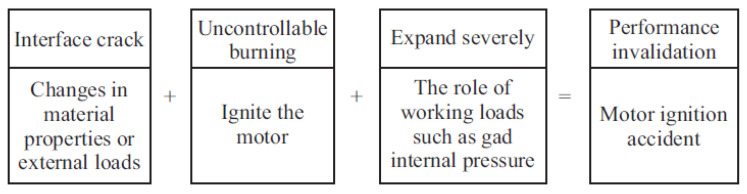
Interfacial bonding failure process of SRM.

The failure of interface performance is interface debonding, that is, macroscopic cracks are produced at the interface. As shown in Figure 8, the failure process of interface performance can be described as follows: local stress concentration or excessive stress caused by material performance changes or external loads at the interface leads to micro-defects such as dislocations or micro-cracks, and finally cracks are formed; Under the action of working load, the crack propagates seriously, and the stable combustion in the engine changes into intense combustion such as deflagration, which leads to the ignition accident of the engine [26]. Whether cracks will propagate or not is an important criterion for interface performance failure. When the crack does not propagate and the interface debonding will only increase the combustion area during the combustion process of propellant, which will cause the change in interior ballistic performance and cause the working load. Under normal circumstances, the engine will not fail to ignite at this time. However, once the crack propagates when the engine works, the burning surface will increase in a very short time, causing deflagration, fire and so on.

**Figure 9:**
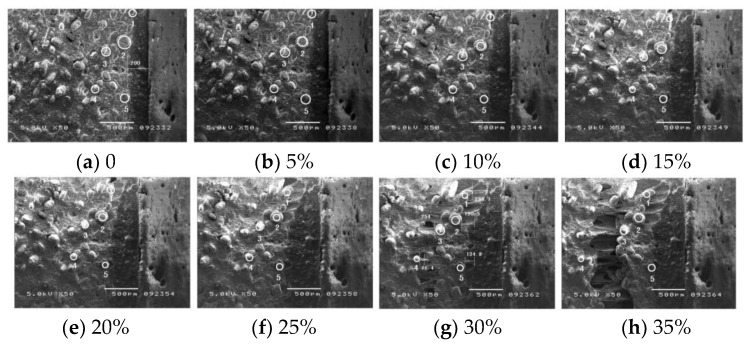
The microscopic failure images obtained by magnification of 50 times using a scanning electron microscope.

Uniaxial tensile test based on bonded specimens is the main method to study interface performance failure [27]. Through microscopic observation of interface debonding process, the change process from debonding defect to complete performance failure can be obtained, and the cognition of interface failure mechanism can be deepened. Li et al. [28] Carried out SEM in situ tensile test on the bonding interface, which showed that the debonding failure process of the interface showed the initiation and propagation of cracks, and the initiation and development of damage near the interface would promote the continuous decline of its bonding performance, as shown in Figure 9. Yang et al. [29] thought that the damage of bonding interface was the result of dehumidification of particles in propellant and debonding of bonding interface through similar methods. Zhou et al. [30] recorded the failure process of bonding interface in the test of double cantilever sandwich beam and thought that the formation and merger of holes near the crack tip was the essence of interface debonding failure. Qiu et al. [31] observed the tensile failure process of interface parts through CCD optical microscope and found that the dehumidification between AP particles and liner is the main reason for the degradation of interface performance. Structural analysis based on finite element numerical simulation is an important method for interface performance failure research, which has the advantages of strong repeatability, good economy and short research period. It is used to determine the stress and deformation of the interface under the environmental load during storage and operation and can provide support for interface failure problems such as interface debonding and crack propagation.

Interfacial debonding is usually caused by local stress concentration or excessive stress, which is closely related to the characteristics of liner materials. Based on the interface bonding model, Jiang calculated the stress and strain distribution of the interface by using the finite element analysis method of interface common nodes. The results show that the increase in elastic modulus of liner will increase the stress concentration near the interface; The larger thickness of the interface layer will enhance the stress concentration near the interface, but will make the failure mode of the interface bonding system tend to cohesive failure, on the contrary, it will improve the bonding quality of the interface. Guo et al. [32] based on three-dimensional finite viscoelastic damage theory, through finite element calculation, it is found that the increase in thermal expansion coefficient and equilibrium modulus of liner will lead to the increase in interface stress at low temperature; Under the action of ignition pressurization load, the increase in initial modulus of liner will also cause the increase in interfacial stress, but the increase in Poisson’s ratio of liner will reduce the interfacial stress. Therefore, liner materials with low modulus, low thermal expansion coefficient and high Poisson’s ratio can effectively reduce the probability of interfacial debonding, and the propagation mechanism of interfacial cracks under working load is another important problem of interfacial performance failure. Conventional finite element method does not converge at the crack tip, can not reflect the displacement mode of the crack tip, and can not describe the problem of interface crack propagation. Therefore, it is necessary to introduce singular crack element and study the interface crack propagation mechanism with the help of stress intensity factor which can reflect the elastic stress field at each point at the crack tip. Xu et al. [33] selected two-dimensional singular crack element to study the interface crack propagation mode under the influence of gas internal pressure, and found that there is a relationship between the stress intensity factor at the debonding crack tip and the crack depth: the stress intensity factor at the crack tip increases with the increase in crack depth; When the crack depth reaches the critical value, the crack will expand rapidly under the influence of the internal pressure of gas, which will affect the normal burning surface of the grain. When studying the crack propagation under the combined action of gas internal pressure and axial overload, Meng et al. [34] used three-dimensional singular crack element to study three propagation modes of opening mode, sliding mode and tearing mode and their corresponding stress intensity factors. It is found that the crack propagates by sliding or tearing under combined loading, and the corresponding stress intensity factor criterion is given. Liu [35] constructed a viscoelastic incremental interface element and found that gas pressurization rate is an important factor affecting crack propagation. When the gas supercharging rate is less than the critical value, the crack will not propagate, which only affects the internal ballistic performance of the engine. On the contrary, the crack will rapidly propagate under the action of the internal pressure of the gas, which makes the combustion in the engine turn into deflagration and leads to serious failure of the engine.

The above research shows that the finite element numerical simulation based on bonded specimens is still an important means to study the interface performance failure and can provide effective support for the study of the mode and mechanism of interface debonding failure. Due to the lack of mature test methods and means to obtain interface performance parameters, the parameters used in numerical simulation generally rely on industry experience, and the consistency between simulation results and real situations needs to be improved. In addition, the experimental study at the specimen level cannot meet the actual needs of engine research, and the finite element analysis at the engine level also lacks the verification of test results. Therefore, it is necessary to explore the method of accurately obtaining interface performance parameters and the test method of interface crack propagation at engine level in the future, so as to improve the failure analysis system of engine interface performance.

#### 2.2.3. Large Deformation of Grain

**Figure 10:**
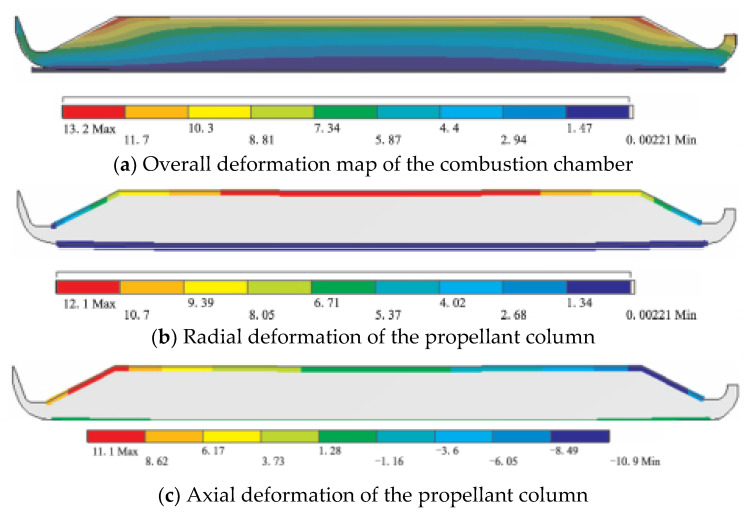
Finite element calculation results of the deformation of each part of the propellant column.

For the grain structure, the main factors affecting its deformation are the volume shrinkage of the grain during curing and cooling, the compression deformation of the grain under working internal pressure, and the irregular structure state of the grain after deformation, and the finite element calculation results of the deformation of each part are shown in Figure 10 [36]. During the whole service period, the external load on the engine is very complicated from the grain filling in the combustion chamber to the ignition and pressure increase in the engine. The first load on the grain is thermal load. Because the solid propellant grain must be solidified, it generally needs to be cooled from solidification temperature to service temperature. For the wall-attached casting grain, it is bonded to the engine shell, and the thermal expansion coefficient of propellant is one order of magnitude higher than that of shell material (the linear thermal expansion coefficient of composite solid propellant is about 10^−4^ °C^−1^, that of double-base propellant is 1.5 × 10^−5^ °C^−1^, and that of steel and glass fiber reinforced plastic is about 10^−5^ °C^−1^), so it is inevitable to produce thermal stress and strain [37]. The most dangerous areas are generally at the inner hole of the straight cylinder section of the grain, the groove surface of the wing and groove, and the interface of insulation-liner-propellant at both ends of the grain. When the propellant solidifies, it will also produce curing heat, which makes the internal temperature of the propellant higher than the controlled curing temperature, and when the propellant solidifies, it will produce curing shrinkage, so the stress generated at this time should be converted and superimposed on the stress generated by curing and cooling. When the initial temperature of curing and cooling is 0 and the stress temperature is *T*_0_, the *T*_0_ of composite propellant is generally 8 °C higher than the curing temperature, and that of double-base propellant is about 15 °C. The zero stress temperature of each propellant grain can be obtained by measuring the internal temperature of the grain during curing and the volume shrinkage of the propellant during curing. In addition, it can be obtained by measuring the change in the inner hole of the round hole engine, and the temperature of the round hole engine is *T*_0_ when the diameter of the middle section of the grain is the same as that of the mandrel. The second load is the acceleration load. The engine will produce axial and lateral acceleration during storage, transportation, in-cylinder launch and rocket flight. When the change in acceleration with time is slow compared with the natural frequency of grain, the acceleration load can be treated as static load; Otherwise, if the acceleration changes with time period, the acceleration load can be treated as impact load. Under the action of slow axial acceleration, the shear stress of the cylinder section of the engine grain is proportional to the diameter, and the displacement of the grain is proportional to the square of the diameter. Therefore, for engines with large diameters, high temperature operation or high acceleration, this negative effect is great. This load may cause cracks in the grain at the inner holes and grooves at both ends of the grain, and even destroy the interface between insulation layer, liner layer and propellant in the middle section. For submersible nozzles and engines with radial slotted grains, the influence of grain sinking on engine interior ballistics should also be considered. Under the action of transverse storage and acceleration, deformation will also occur. In order to prevent the shell and grain from deforming too much during storage, the engine can be rotated regularly. The third kind of load is mainly the working internal pressure of the engine or the protective pressure during storage. This kind of load exists all the time from storage state or ignition to flameout, and the engine shell and grain will deform under the action of internal pressure, and the gas in the inner channel will be blocked after the grain deforms greatly.

If the nozzle or gas passage is blocked, it will inevitably cause gas choking, which will lead to a sharp rise in the pressure in the combustion chamber and cause explosion. Judging from the structure of the engine, there are two possibilities for gas choking: grain breakage and initial deformation, elongation and blockage of gas passage [38]. The breakage or rupture of grain is caused by the mechanical properties of grain under the action of external load, which can not meet the design requirements.

The damage property of propellant is to study the ultimate stress–strain of propellant under the condition of maintaining structural integrity and whether the structure of propellant grain is damaged when propellant is subjected to various loads. Only when the ultimate stress and strain of propellant are greater than the stress and strain produced by practical force, the propellant grain is safe and reliable. According to the numerical analysis results of 100 G and 50 G impact tests and the theoretical calculation of a certain unit on the grain subjected to flight overload, the maximum stress is close to the ultimate stress of the grain. Therefore, the possibility of damage of the engine grain after primary and secondary loading cannot be ruled out. Through the analysis of field data, it can be seen that when the grain stretches about 4 mm, erosion combustion will occur, and when the grain stretches about 8~12 mm, the maximum pressure in the combustion chamber will sharply increase the bearing limit of the shell; From the impact test results of 100 G and 50 G, the elongation deformation of this grain is relatively large [39]. Therefore, it is of great significance to consider the typical fault of grain elongation and deformation.

#### 2.2.4. Grain Aging

**Figure 11:**
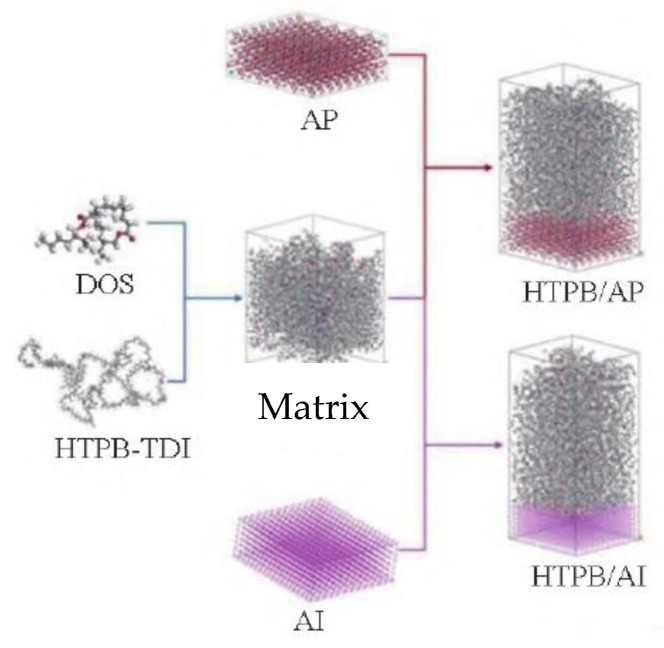
Microscopic structure diagram of solid propellant [40].

The main components of composite solid propellant grain are high molecular polymer. The schematic diagram of Its microstructure is shown in Figure 11 [40]. Therefore, the aging of grain is very complex, and the change in original performance of propellant is often the result of interaction between external physical aging and internal chemical processes. Chemical aging refers to the change in propellant performance caused by continuous chemical change during storage after the completion of processing and curing cycle; Physical aging refers to the change in propellant properties caused by some physical factors (such as crystal change, phase change, component migration, stress, environmental humidity, etc.) during storage. Aging phenomenon is the result of comprehensive influence of physical and chemical factors.

The safety factor calculated from the original mechanical properties can not correctly reflect the influence of these interactions. In the process of aging research, people pay most attention to chemical reactions including thermal degradation reactions, but in fact, some physical reactions may make the properties of propellants age with time, and this aging has nothing to do with chemical changes. For example, the gradual loss of volatile matter will lead to the hardening and tensile strength reduction in propellant, and will also reduce the volume of propellant slightly, and finally lead to higher stress and strain levels in shell-bonded propellant grain. In addition, many fine cracks gradually expand under stress, which reduces the strain durability of propellant or bonding interface. When the crack size reaches the critical state, the material fails [41]. Table 1 lists the influencing factors that cause the grain performance to decline during the aging of solid motors.

Therefore, the main influencing factors of grain aging are as follows:

(1) Effect of temperature

**Figure 12:**
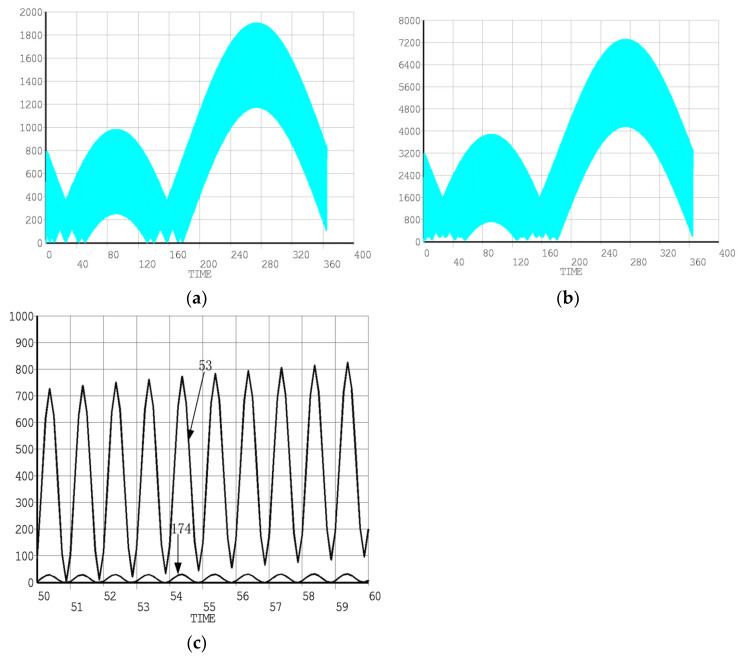
Analysis of the aging performance of solid propellants under different temperatures [42]. (**a**) The equivalent stress-time curve at node 53; (**b**) The equivalent stress-time curve at node 174; (**c**) Node 53, 174 in the stress comparison chart over a period of 50 to 60 days.

Temperature is the most important factor affecting various storage environments. With the increase in temperature, the degradation or crosslinking speed of binder in propellant is accelerated, the thermal decomposition speed of oxidant is increased, and the bonding force between plasticizer and binder is relaxed and the migration is accelerated; When the temperature drops to a certain value, polymer crystallization may occur. The stress variation curves of the key points of the solid propellant were calculated through finite element analysis, as shown in Figure 12 [42]. After leaving the factory, the solid rocket arrives at the user after one month’s transportation. This process is in June and July. The weather is hot, and the environment of the grain is equivalent to high temperature and heat preservation. According to the time-temperature equivalence principle, a short-term temperature rise is equivalent to low temperature and long-term storage, which means accelerated aging. Therefore, the mechanical properties of one-month transportation are quite different from those of one-month normal temperature storage.

(2) The influence of humidity

**Figure 13:**
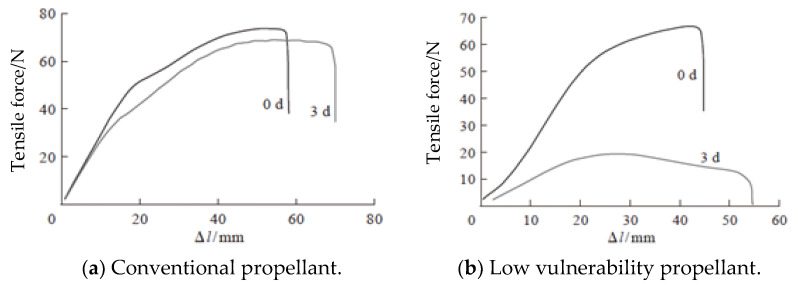
The tensile mechanical property curve of the solid propellant after 3 days of moisture absorption [43].

Humidity is another important factor affecting the mechanical properties of composite solid propellant, as shown in Figure 13 [43]. There are three main sources of water in propellant. One is brought in by incomplete drying of propellant raw materials; the second is the chemical reaction between propellant components; the third diffuses from the atmosphere and enters the propellant. During the transportation of solid rocket, from a certain unit to the user passing through some southern provinces, and the relative humidity in these provinces is very high in June and July, which may cause the following results: the hydrolysis and chain breaking of binder, causing the propellant to soften; moisture forms a low modulus “coating layer” on the surface of oxidant to reduce the binding force between oxidant and binder, which can produce “dehumidification” phenomenon under the action of low stress and lead to the decrease in mechanical properties; and moisture diffuses to the interface of propellant/liner/insulation, thus reducing the bonding force of the interface.

(3) Vibration effect

**Figure 14:**
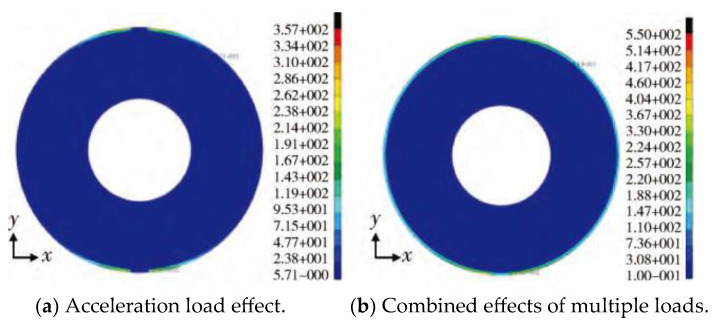
Under the action of vibration, the force response graph of SRM [44].

**Figure 15:**
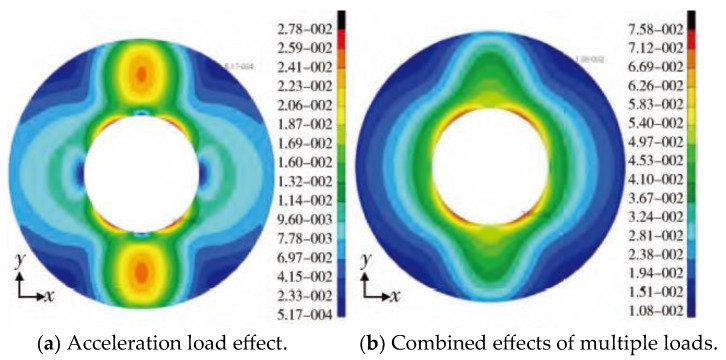
Mechanical response of solid propellants under the action of road surface vibration [44].

For an ideal elastic body, after the action of alternating force, due to the action of elastic restoring force, it will vibrate at the equilibrium position, and its energy will be used for elastic deformation, and its amplitude has nothing to do with time, showing undamped free vibration. For viscous liquids, the kinetic energy is completely absorbed and converted into heat energy and dissipated, and the amplitude decays rapidly with time, showing a typical damping motion. For viscoelastic body, it is between them, one part is used for elastic deformation, the other part is converted into heat energy and lost, and the amplitude decreases gradually with time, which is damped vibration. According to the data provided, the road conditions of solid rockets are bad during transportation, so the influence of vibration caused by road quality problems on missiles cannot be ignored, as shown in Figure 14 and Figure 15. Under the action of vibration, the force response graph of SRM [44]. Long-term vibration will convert part of energy into heat energy. Composite solid propellant is a poor conductor of heat, and a large amount of heat energy will increase the temperature of grain. Because the outdoor temperature during transportation is higher than normal temperature, the mechanical properties of grain will decrease faster.

### 2.3. Failure Analysis of SRM Grain

**Figure 16:**
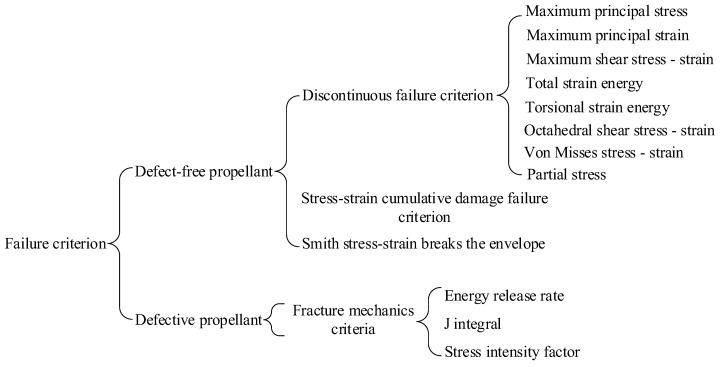
Common failure criteria of SRM grain.

The failure mode of SRM is generally constructed by its failure criterion, and the failure mechanism and failure criterion of grain are a research hotspot. At present, there is no completely reliable failure criterion to follow, and the strength theory of metal materials can still be used, such as maximum stress theory, maximum strain theory, maximum shear stress theory, maximum shear strain theory and maximum strain energy theory [15]. At present, the common failure criteria of solid propellants, as shown in Figure 16, are derived according to the classical failure theory of plastic brittle metals and considering the viscoelasticity of propellants. Each criterion specifies some definite function of stress field or strain field, and the limit value of this function, that is, the right-hand term of the criterion, needs to be determined by experimental method. When the function value exceeds its limit value, the propellant will yield, destroy or fracture. The failure criterion of grain is a very complex problem, which involves many factors such as multidirectional stress state, loading history, strain rate, temperature and humidity aging, etc. There are still many problems to be studied. Therefore, the failure criteria summarized in this paper are mainly determined according to engineering experience, but they are applicable in engineering practice.

The engine shell is the main load-bearing component of the engine, and the grain is bonded with the shell through the coating (or insulation layer) to ensure that the structural integrity of the grain remains intact under various possible loads and environmental conditions. The development practice of engine proves that the development direction of propellant mechanical properties is to greatly improve elongation under appropriate strength [45]. On the one hand, the proper requirement of propellant modulus is that the grain should not deform too much, which not only affects the interior ballistics, but also may block the nozzle and lead to accidents. On the other hand, it is mainly to make the grain have the ability to bear axial or transverse overload, which must be borne by the strength of the grain itself, including the bonding strength between propellant and coating, coating and insulation, coating, insulation and shell. In addition, the engine bears various loads, such as curing and cooling, storage, transportation, service processing, ignition, and launch. Therefore, when analyzing the structural integrity of the engine under various loads and environmental conditions, the failure criterion should adopt a mixed type [46,47,48].

#### 2.3.1. Failure Analysis Based on Maximum Strain

In the integrity analysis of grain inner surface, the maximum strain criterion is often used when the infinitesimal body is in unidirectional or biaxial stress state. Wang Yuanyou mentioned in the article “Design of SRM” that at low temperature, the stress and strain on the inner surface of the grain are the maximum. At low temperature, the grain has the maximum ability to bear stress and the minimum ability to bear strain. Therefore, it can be considered that the crack on the inner surface of grain at low temperature is caused by the maximum circumferential strain $\varepsilon_{\theta }$ exceeding the maximum stretching elongation $\varepsilon_{m}$. In addition, when the grain is subjected to temperature load and working pressure load, it is more appropriate to take elongation as the criterion; This is because under the working pressure load and temperature load, because the elastic modulus of the engine shell is very large, it is the main bearing component of the engine, and the grain can keep its integrity as long as it deforms without cracks and debonding, thus making the grain structure work reliably [49]. Therefore, the conditions for no cracks on the inner surface of the grain are as follows [50]:

(3) $$D=\varepsilon_{\theta }\;\left(r=a\right)/\varepsilon_{m}\;<1$$

#### 2.3.2. Failure Analysis Based on Octahedral Shear Stress or Shear Strain

Propellant is a high molecular polymer with polymer binder as matrix, oxidant and aluminum powder as solid filler, and contains a large number of solid particles. The binder used in the coating is usually the same as that of propellant. Therefore, the coating and propellant belong to viscoelastic materials, and the engine is in a multi-directional stress state under the action of internal gas pressure or other loads. Because of the good toughness of propellant, it is reasonable to adopt octahedral shear stress or octahedral shear strain as criterion [51]. In addition, because the engine shell is rigid and is the main bearing component of the engine, the grain can keep integrity as long as it can deform without cracking and debonding, so it is generally considered that the equivalent strain criterion can be used to judge the structural integrity of the grain, that is, octahedral shear strain or Von Mises strain is generally used as the failure criterion of the grain, i.e.,

(4) $$\gamma_{8}\leq \frac{\gamma_{8m}}{n}$$

where $\gamma_{8m}$ represents the critical value and *n* denotes the factor of safety, the octahedral shear strain can be expressed as

(5) $$\gamma_{8}=\frac{2}{3}\sqrt{{\left(\varepsilon_{x}-\varepsilon_{y}\right)}^{2}+{\left(\varepsilon_{y}-\varepsilon_{z}\right)}^{2}+{\left(\varepsilon_{z}-\varepsilon_{x}\right)}^{2}+6(\varepsilon_{xy}^{2}+\varepsilon_{xz}^{2}+\varepsilon_{yz}^{2})}$$

In the case of simple stretching, $\varepsilon_{y}=\varepsilon_{z}=-\nu \varepsilon_{x}$, $\varepsilon_{xy}=\varepsilon_{yz}=\varepsilon_{zx}=0$, the relationship between the critical value of $\gamma_{8}$, $\gamma_{8m}$ and the maximum stretching elongation $\varepsilon_{m}$ is

(6) $$\gamma_{8m}=\frac{\sqrt{8}}{3}(1+\nu )\varepsilon_{m}$$

The Von Mises strain expression is

(7) $$\varepsilon_{V}=\frac{\sqrt{2}}{3}\sqrt{{\left(\varepsilon_{x}-\varepsilon_{y}\right)}^{2}+{\left(\varepsilon_{y}-\varepsilon_{z}\right)}^{2}+{\left(\varepsilon_{z}-\varepsilon_{x}\right)}^{2}+6(\varepsilon_{xy}^{2}+\varepsilon_{xz}^{2}+\varepsilon_{yz}^{2})}$$

(8) $$\gamma_{8}=\sqrt{2}\varepsilon_{v}$$

It can be seen that the Von Mises strain criterion is essentially equivalent to the octahedral shear strain criterion:

(9) $$\varepsilon_{V}\leq \frac{\varepsilon_{Vm}}{n}$$

Among them, n is the safety factor,

(10) $$\varepsilon_{Vm}=\frac{2}{3}(1+\nu )\varepsilon_{m}$$

Poisson’s ratio of grain is 0.495~0.498, so it can be seen from Equation (8) that it is approximately equal to the maximum tensile elongation, which is also convenient to use Von Misses strain as the judgment criterion for structural failure analysis of SRM [14].

#### 2.3.3. Failure Analysis of Interfacial Debonding of Grain

There are many interfaces in SRM, and interface debonding is another common failure mode. Under the action of external load, when the interface tearing stress or shear stress exceeds the interface tearing strength and shear strength, debonding failure may occur at each bonding interface (shell/insulation layer, insulation layer/cladding layer, cladding layer/propellant bonding interface), and its criterion is as follows [52]:

(11) $$\sigma_{n}\leq \left[\sigma_{n}\right]\;or\;\tau_{t}\leq \left[\tau_{t}\right]\;$$

where, the normal tensile stress $\sigma_{n}$ of the interface is its allowable value $\left[\sigma_{n}\right]$; $\tau_{t}$ is the tangential shear stress of the interface, which is its allowable value $\left[\tau_{t}\right]$.

#### 2.3.4. Failure Analysis of Structural Stiffness of Grain

Solid propellant is a kind of rheological material, and the geometric surface of the grain will change during the storage of the engine. When this deformation exceeds a certain range, the geometric shape of the burning surface of the grain will change or block the gas passage, thus affecting the interior ballistic performance of the engine [53]. The criterion is

(12) $$u_{i}\leq \left[u_{i}\right]$$

Among them, the deformation $u_{i}$ in a certain direction of the dangerous point is its allowable value $\left[u_{i}\right]$ [14].

#### 2.3.5. Failure Analysis of Crack Instability of Grain Structure

Crack instability propagation means that the crack of propellant grain exceeds the maximum allowable crack length, which makes the crack instability propagation when the engine is ignited and launched, resulting in launch failure. Because propellant is a linear viscoelastic material at working temperature, stress intensity factor (*K* criterion), *J* integral (*J* criterion) or energy release rate (*G* criterion) can be generally considered as the crack propagation criterion of grain., i.e., [54]

(13) $$K_{ig}\leq K_{igC},\;J_{g}\leq J_{gIC}\;\;or\;\;G_{g}\leq R_{gIC}$$

where *K_ig_* (*i* = I, II, III), *J*_g_ and *G_g_* are the stress intensity factor, *J* integral and energy release rate at the crack tip of grain, respectively; *K_igC_*, *J_gIC,_* and *R_gIC_* are the fracture toughness, critical *J* integral and propagation resistance of grain crack, respectively.

#### 2.3.6. Failure Analysis of Interfacial Crack Propagation of Grain

The unstable propagation of interface crack means that the length of debonding crack at the interface of shell-insulation layer, insulation layer-cladding layer or cladding layer-propellant exceeds the maximum allowable value, which makes the interface crack unstable and propagate when the engine is ignited and launched, resulting in the engine penetrating fire [55]. In this case, the stress intensity factor, *J* integral or energy release rate of interface crack can be considered as the propagation criterion of debonding crack [14].

(14) $$K_{i}\leq K_{iC},\;J_{i}\leq J_{iIC}\;or\;\;G_{i}\leq R_{iIC}\;$$

where *K_i_* (*i* = I, II, III), *J_i_* and *G_i_* are the stress intensity factor, *J* integral and energy release rate at the interface crack tip, respectively; *K_iC_*, *J_iIC_* and *R_iIC_* are fracture toughness, critical *J* integral and propagation resistance of interface crack, respectively.

#### 2.3.7. Failure Analysis Based on Cumulative Damage

From casting to ignition, the grain experiences various loads. For the grain, every load will cause certain damage [56]. The damage caused by these loads together is called cumulative damage. The cumulative damage of grain can be estimated according to the cumulative damage theory of metal materials proposed by Miner.

Let *N* be the number of cycles when the specimen is damaged by the first-order stress; *N_i_* is the number of cycles of the specimen subjected to the first-order stress. Mainer pointed out that if the specimen is subjected to *n*-order stress, when

(15) $$\sum_{i=1}^{n}\left(N_{i}/N_{Fi}\right)=1$$

the specimen will fail [14].

According to Williazns and Miner’s theory of linear superposition of damage mentioned above, it is concluded that the cumulative damage on the inner surface of grain is

(16) $$\sum D_{i}=\sum_{i=1}^{n}\frac{\varepsilon_{i}}{\varepsilon_{mi}}$$

At that time, when $\sum D_{i}=1$, the inner surface was damaged and failed [57,58,59].

## 3. Reliability Analysis of SRM Grain

### 3.1. General Overview

**Figure 17:**
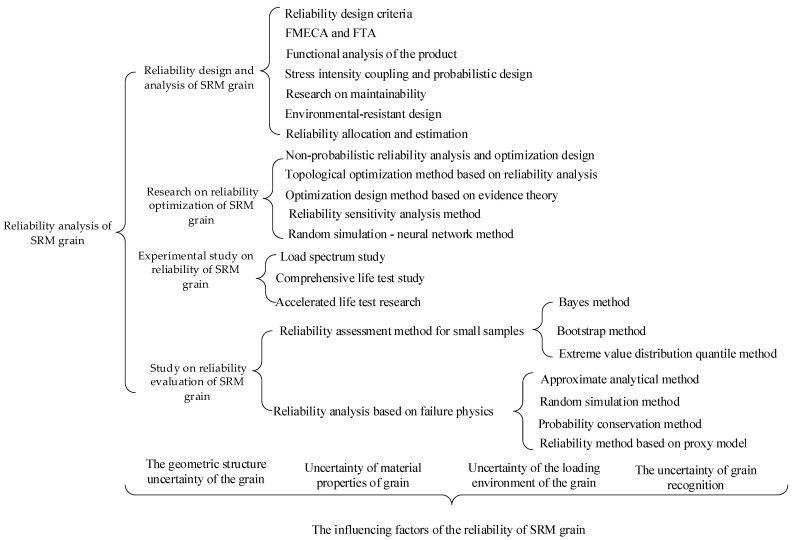
The reliability analysis method and influencing factors flowchart of SRM grain.

Uncertainties can be seen everywhere in the analysis process of SRM grain. If uncertain parameters are ignored, the structural integrity of engine grain and the accuracy of reliability evaluation will be affected. Due to the influence of parameter uncertainty, the design of charge structure will eventually fail, which will lead to huge losses, as shown in Figure 17 [60]. In the engineering field, uncertain factors always exist. In recent years, the probabilistic reliability method has become a common method to deal with uncertain factors, including the probabilistic reliability evaluation of SRM grain. In traditional grain structure design, uncertain parameters are usually treated as definite values, even some parameters determined by experiments are uncertain, and the parameters determined by experiments are generally treated as mean values and then taken into the model for structural analysis and design. With the improvement of numerical calculation ability and the development of the reliability theory, especially the application of finite element method, many engineering problems have been solved by breaking through the obstacles of material physical parameters change, irregular geometric boundary conditions and complex displacement constraints. Therefore, the structural design and reliability analysis of grain based on uncertain parameters have also been developed to some extent. The flowchart of the reliability analysis method and influencing factors for SRM grain is as follows:

### 3.2. Reliability Design and Analysis of SRM Grain

The following problems need to be considered in the reliability design and analysis of solid propellant grain: (1) The distribution of all kinds of fault problems is unknown, so it is difficult to unify the failure criterion methods. (2) There are many failure modes, complex influencing factors and high correlation. (3) There are few fault data of grain structure, and the degree of dispersion is large. (4) It is obviously affected by load. (5) The launching working conditions and environmental conditions are complex and extremely harsh. (6) The limitation of processing technology leads to great difference in product performance. (7) The limitation of test technology and test conditions leads to the inability to guarantee adequate test. Therefore, in the reliability analysis and design of grain structure, attention should be paid to making use of the past engineering practice experience to formulate design criteria to guide the reliability design of grain structure [18]. At the same time, we should pay attention to the analysis and research of failure mode and adopt improvement scheme to improve the reliability of grain through the research of failure mechanism.

Based on the above technical characteristics, scholars at home and abroad have carried out a large number of reliability design and analysis studies [61,62,63,64], including the following aspects: (1) On the basis of fault analysis, the reliability design criteria have been formulated, and the experience of reliability design and reliability measures has been organized and standardized; (2) Through the analysis and research of fault modes, various analysis methods such as FMECA and FTA have been put forward. (3) Considering the discreteness of product data, the functional analysis of product is carried out. (4) Carry out stress intensity interference and probability design of parts, so as to measure the safety margin of parts. (5) Carry out maintainability research from product design. (6) Aiming at vibration, impact and product sealing, a large number of environmental resistance design studies have been carried out. (7) Carry out research on product reliability allocation and prediction. (8) Correlation analysis is carried out for multiple failure mode analysis. (9) A large number of technical foundations of reliability test have been accumulated, including life test, accelerated life test, reliability appraisal and acceptance test, etc.

### 3.3. Research on Reliability Optimization of SRM Grain

Solid motor grain structure is complex and has many design parameters. It is extremely difficult to determine multiple design parameters simultaneously by a simple reliability design method. Structural reliability optimization must be carried out in combination with optimization theory. Mechanical reliability optimization design originated from Feigen and Hilton’s research on the design problem of minimizing structural weight under safety conditions at the end of the 20th century. On the basis of deep development of mechanical reliability theory, great progress has been made in solving reliability optimization problems of mechanical structures based on the reliability theory abroad [65,66,67]. Elishakoff et al. [68] proposed to use uncertain convex set model to describe numerical problems and practical engineering problems and carry out non-probabilistic reliability analysis and optimization design. Dimitrov et al. [69] put forward a model factor correction method for the reliability analysis of composite structures based on finite element analysis technology and applied it to reliability analysis and optimization design of turbine blades. Eom et al. [70] put forward a topology optimization method based on reliability analysis on the basis of standard response surface method and applied this method to reliability optimization design of three-dimensional structures, which improved the efficiency of reliability optimization design. Mourelatos et al. [71] put forward an optimization design method based on evidence theory, which can quickly search the optimal neighborhood and determine the effective constraints, thus introducing evidence theory into reliability optimization design method. Alyanak et al. [72] developed a reliability optimization design method based on evidence theory by using a gradient mapping method.

In addition, because the reliability sensitivity analysis method plays an important role in the reliability optimization design of mechanical structures, many scholars put forward very effective reliability sensitivity analysis methods, which have been applied in practical engineering fields. Rackwitz [73] put forward the concept of reliability sensitivity to random variables. After the concept of sensitivity was put forward, Bjerager [74] studied the first-order reliability sensitivity problem. Karamchandani and Cornell [75] put forward a reliability sensitivity analysis method based on quadratic second moment. Melchers proposed a fast approximate solution of reliability sensitivity analysis method based on Monte Carlo simulation [76]. On the basis of establishing reliability topology optimization model, Zhan et al. [77] applied level set method to reliability optimization design of compliant mechanism. Meng et al. [78] used subset simulation approximation to approximate reliability and sequential optimization solution model method to optimize the reliability design of hydraulic transmission mechanism. Based on the reliability analysis of extreme response surface method, Zhang et al. [79] established the mean value model of reliability optimization design of flexible mechanism and solved the model by using the first-order second moment method. Han et al. [80] combined mean model and probability model with decomposition and coordination theory and put forward mean-probability decomposition and coordination method for reliability optimization design, and applied this method to reliability optimization design of flexible manipulator. Zhang et al. [81] organically combined stochastic simulation method with artificial neural network technology and put forward stochastic simulation-neural network method for mechanical reliability optimization design, which provided a new method for optimization design field. Miao et al. [82] proposed a three-step analysis method considering the initial deformation of grain based on the existing numerical calculation methods. The sensitivity of grain structural integrity to axial deformation and radial deformation of shell was analyzed by using the full factor experimental design method, and the estimated pressure was given. Then, the Evol optimization algorithm was used to optimize an airfoil column engine based on the estimated pressure.

Generally speaking, reliability optimization technology has been developed in the field of structural optimization, and its application in SRM grain structure has not been reported. Therefore, there is still a lot of in-depth work to be carried out in the reliability optimization design of grain structure.

### 3.4. Experimental Study on Reliability of SRM Grain

With the increasing requirement for the reliability of SRM grain, test technology plays an important supporting role in verifying the research and development objectives of high-reliability and long-life products. At present, it is difficult to complete the reliability evaluation of products in an effective time by traditional life test technology. In addition, due to the accelerating iterative speed of product renewal, higher requirements are put forward for the rapid acquisition of product life indicators. Therefore, carrying out reasonable and effective life tests has been widely valued in the field of reliability test engineering. At present, researchers have carried out a lot of research work on life test and obtained relevant research results [83,84,85].

In the study of load spectrum, because SRM works under harsh conditions of high temperature and high pressure, the fault categories and failure modes are complex. From the fault statistics of SRM, it is found that grain fault is the main fault and the main threat to engine safety. Whether the engine load spectrum can truly reflect the actual use of the engine directly affects the engine life evaluation results. Chinese military standard GJB241A-2010 [86], American military standard MIL-E-5007D [87] and structural completion outline MIL-HDBK-1783 [88] clearly point out that load spectrum is the basis for stress analysis of parts, structural design criteria, durability test of parts and whole machine and life analysis. Load spectrum runs through the whole process of product design, test and life determination. Through the formulation of product load spectrum, the reliability of life evaluation can be effectively improved, thus ensuring its use safety. The data processing method of load spectrum mainly adopts cycle counting method, among which rain flow counting method is widely used in engineering. The subcycle determined by the rain flow counting method corresponds to the stress–strain hysteresis loop of the material and can reflect the low cycle fatigue damage. At present, rain flow counting method is commonly used to count low cycle fatigue at home and abroad, which is used for fatigue life estimation and fatigue test load spectrum compilation. It has achieved effective development in engineering applications and achieved good engineering application effects [89].

In the research of complete life test, with the development of mechanical products towards complexity, miniaturization and precision and the demand of modern industry, the reliability demand of mechanical products is increasing day by day. Whether the mature reliability life test models and test methods of electronic products can be applied to the reliability life test of mechanical products has always been the focus of discussion on reliability life test [90,91,92]. The failure probability density distribution of every part of the whole machine is proposed by researchers, which can be approximately considered as exponential distribution, and it is too conservative to use exponential distribution to evaluate the life of mechanical structure. However, most tests show that the failure probability density distribution of mechanical products mostly obeys lognormal distribution or Weibull distribution. Because the grain structure is complex and the matching accuracy is high, the reliability of the whole machine is required to be high in the harsh environment such as high temperature, high pressure and strong load in practical work. Traditional reliability tests, such as full life test, are difficult to test and consume a certain amount of manpower and material resources. Therefore, in order to improve the efficiency of product test and the economy in the process of product development, accelerating life test research has become the main research direction at present [93].

The accelerated life test of SRM grain is compared with the accelerated life test technology to check the reliability of products. The durability test method of overspeed (115%) and overpressure test is adopted. Through this test, it shows that the grain can meet the life index requirements of solid rocket under severe working conditions. From 1980s to now, Russia still refers to the published and implemented accelerated life test method guide to carry out relevant tests. At present, China still refers to the Russian test technology system and adopts accelerated life test to carry out the test assessment of grain. The failure mode of grain is the main basis for selecting accelerated life model. When choosing acceleration models, we mainly select mature models such as the Arrhenis acceleration model, inverse power law acceleration model, Maine rule, Erin model and comprehensive test model. After a large number of actual product tests and comparative analysis and combined with typical faults of grain, it is considered that the Arrhenis model combined with time-temperature equivalent model can be selected for accelerated test research for aging failure mode [94,95,96]. In addition, when applying the model, there is still a problem that is whether there is a superposition relationship (product relationship or other relationship) among multiple stresses, which is difficult to determine at present. In the accelerated life test of grain, it is necessary to compile the accelerated equivalent test outline, which must specify the following working conditions in advance: first, the equivalent working condition of maintaining the accelerated test state, that is, the working condition of the total section of rocket equipped with engine; The second is to maintain continuous working conditions such as curing and cooling, temperature cycle, vibration, acceleration and ignition pressure building.

In recent years, scholars at home and abroad have carried out experimental research on grain life assessment. In order to predict the storage life of vertical storage solid motor grain, Wang et al. [97] comprehensively considered the influence of accelerated aging and measured load and carried out accelerated aging test of propellant at high temperature, and obtained the change law of propellant elongation. Based on the variation law of elongation and strain with time, the engine life is predicted. Wang studied the aging properties of the coating through thermal accelerated aging test and tensile mechanical properties test, analyzed the influence of aging temperature and aging time on the mechanical properties of the coating, established a life prediction model based on characteristic indexes and a thermal aging constitutive model of EPDM rubber, and accurately predicted its storage life [98]. Based on three-dimensional viscoelastic stochastic finite element method and accelerated aging test of solid propellant at high temperature, Zhou studied the influence of curing strain on long-term storage life of engine, and the influence level of propellant performance parameters and load conditions on Von Mises strain of grain, and carried out research on aging mechanism of HTPB propellant and engine life prediction under constant strain [42]. Tang et al. [99] statistically analyzed the aging test data and obtained the variation law of digital characteristics of solid propellant performance parameters with storage time. The mean and standard deviation of structural response of an SRM grain were calculated by using three-dimensional viscoelastic response surface stochastic finite element method, and the structural reliability of an SRM grain in different storage periods was analyzed, and its probabilistic storage life was predicted. Cao et al. [100] took a composite solid propellant grain as the research object and carried out high temperature accelerated aging test. By studying the storage properties of HTPB propellant under high temperature accelerated aging conditions, the variation laws of various storage properties with aging time were obtained, the aging mechanism of HTPB propellant under high temperature accelerated aging conditions was analyzed, and the life of HTPB propellant grain was predicted. Peng et al. [101] studied the creep characteristics of long-term vertical storage solid motor grain under the influence of aging and vibration. Based on generalized Kelvin model, a creep constitutive model considering aging and damage factors was constructed. Through temperature and stress accelerated creep test, high temperature accelerated aging test and reciprocating tensile damage test, the influence law of aging and damage on creep is obtained, and the vertical storage life of a certain engine is calculated. Based on the idea of material high-throughput test, Wang put forward a high-throughput accelerated test method for solid propellant, developed a high-throughput accelerated test system for propellant, carried out high-throughput accelerated test on a certain HTPB propellant sample, and established a propellant relaxation modulus aging model based on high-throughput test. The research results and conclusions can support the rapid prediction of storage life of solid propellant [102].

To sum up, the key test technology of reliability life is very important to realize the development of high reliability and long-life grain. At present, there is still no effective method for the study of load spectrum of grain in China, and there is still a gap between the related life and reliability test technology and test conditions compared with those in foreign countries, so the test methods of acceleration model, acceleration ratio and acceleration factor of accelerated life test with wider applicability can not be completely put forward.

### 3.5. Study on Reliability Evaluation of SRM Grain

In the field of reliability evaluation, due to the economic and structural complexity of products, most of the research is based on small sample data to complete the reliability evaluation. The research of reliability evaluation method in the case of small sample can reduce the number of tests and the consumption of manpower, material resources and financial resources, which has important theoretical value and practical engineering significance. At present, the statistical inference analysis and evaluation method of small samples has been paid more and more attention. Foreign statistical methods for small sample ordered consistent decision-making, the Bayes method, the Bootstrap method and, based on statistical theory, the support vector machine method [103]. In 1980s, the US military carried out the reliability evaluation of Pershing II missile based on the Bayes small sample theory. In 1984, the US military proposed that the sequential analysis method or Bayes small sample theory should be used to carry out destructive tests of expensive systems. After that, Willits and Dietz carried out the reliability analysis of the system composed of binomial distribution and exponential life units, respectively. Springer and Thompson use Mellin Transform to obtain a strict lower confidence limit method [104]. Because the Mellin transform method is only suitable for simple reliability structures, such as series and parallel systems, and the expression is quite complex, it is difficult to realize in engineering. Therefore, Dietrich et al. put forward Chebyshev polynomial approximation method. Winterbottom put forward Cornish–Fisher expansion method for reliability analysis [105]. According to the characteristics of pre-evaluation of weapon equipment test analysis, Zhang has made some achievements in the research of the small sample reliability evaluation method [106]. Fu also studied the reliability method of statistical inference with small samples [107]. In the research of reliability evaluation methods for small samples, the Bayes method has made many achievements. Jiang et al. carried out reliability evaluation for small samples based on Bayes and achieved high accuracy results [108].

The research on statistical inference theory of small samples began in 1960s in China, especially after the 1980s, and great progress has been made, especially in the field of test evaluation and appraisal of weapon systems. A series of statistical inference methods of small samples have been put forward, including the Bayes method, Bootstrap method, Bayes Bootstrap method, compatibility test method of small samples, extremum distribution percentile method and so on. The Bootstrap method is a general method proposed by Efron to approximate the distribution of estimated values of complex statistics, which is considered as one of the important achievements in the field of statistics [109]. Hearst et al. [110] put forward a new learning algorithm-support vector machine on the basis of statistical theory, which realized the inductive principle of structural risk minimization, and had complete statistical theoretical basis and excellent learning performance. Later, support vector machine was used to approach implicit limit state equations in the case of small samples, and Monte Carlo simulated the reliability of the calculation system [111]. Feng et al. studied the reliability evaluation method of success or failure products under the condition of small sample [112]. Liu summarized the acquisition and processing of prior data, including simulation test information, unit and subsystem test information, similar system information, unit and subsystem test information in different environments, historical information and expert opinion information, and studied Bayes evaluation of common reliability unit models [113].

In the research of the Bootstrap method, aiming at the problem that the maximum likelihood estimation MLE method will produce large errors in solving distribution parameters in the case of small samples, Zhang et al. [114] put forward B-MLE method based on the idea of Bootstrap data expansion, which reduces the error of parameter estimation. In order to make full use of the information of small sample failure data and zero-failure data to realize accurate evaluation of product reliability, Wang took the product whose life obeys Weibull distribution as an example, and studied the reliability evaluation methods of small sample and zero-failure data from the reliability evaluation of small sample, zero-failure data and mixed test data to obtain accurate evaluation results [115]. Ge et al. [116] put forward a Bootstrap data expansion method based on support vector regression to solve the reliability evaluation problem of aero-engine with small sample characteristics. By establishing and training the support vector regression model, the input set is constructed by neighborhood sampling method, and the expanded samples are obtained from the trained model. The results show that the expanded samples obtained by this method are closer to the real distribution than the traditional Bootstrap method, and the sample value interval is effectively expanded. Picheny et al. [117] put forward the Bootstrap method to estimate the reliability. By biasing the system response distribution, the performance of the Bootstrap method is compared with other estimation methods. The relationship between conservative evaluation and normal distribution and lognormal distribution is discussed. The influence of sample size and target failure probability on the estimation quality is analyzed, and a conservative but not too conservative estimation is obtained. Finally, this method is applied to the reliability optimization of composite plates under thermal load. Marks et al. [118] proposed a Bootstrap method to find the empirical system life distribution for coherent systems modeled by reliability block diagram, and derived the non-distribution expression of deviation related to the Bootstrap method, which was used to estimate the average system life of parallel and series systems with statistically the same components. At present, computer simulation methods are widely used in reliability evaluation of complex systems. Martz et al. [119] compare classical methods, Bayes methods and Monte Carlo methods, in system reliability confidence interval estimation. Cheng et al. [120] put forward two active learning algorithms based on Bayesian support vector regression model, which, respectively, estimate the large failure probability and small failure probability of complex structures under limited model evaluation conditions. According to Bayesian network prediction and analysis, Li et al. [121] determined the reliability characteristics of offshore floating wind turbines, such as failure probability, failure rate and mean time between failures, and modeled and analyzed the reliability of offshore floating wind turbines. Therefore, the Bayes evaluation theory plays an important role in the reliability evaluation of SRM grain, and its application scope will be wider and wider.

The purpose of the above research is to evaluate the reliability index and life distribution of products based on failure sample data. Another research idea is to estimate the reliability and life index of products by combining physical failure mechanism modeling with probabilistic safety analysis. This kind of research needs to establish the physical mechanism model of each main failure mode and the probability model of random parameters such as material, processing, manufacturing and service load. One of the key problems of reliability technology based on failure physics is failure mechanism modeling for failure modes. Aiming at the typical faults such as deformation aging and fracture of solid motor grain each fault mechanism model is established, and the fault is reproduced by model simulation. Based on the mechanism model the limit state equation of failure criterion is established. Usually, no matter how fine the mechanism model is established, it is subject to complex physical mechanisms, and there will be a certain gap between its prediction and the real situation. The mechanism model can be verified and confirmed in combination with experimental data. If necessary, it is necessary to adopt deterministic or uncertain model correction methods to correct or infer the model errors and model parameters of the mechanism model.

The methods of reliability evaluation based on failure mechanism modeling can be divided into four categories: (1) Approximate analytical methods, which mainly include first-order moments and second-order moments. These methods have high computational efficiency and are generally used for problems with low nonlinearity. (2) Stochastic simulation methods, including the importance sampling method, subset simulation method and line sampling method, are suitable for high-dimensional and small probability problems, but the calculation cost is generally high. (3) Probability conservation methods, including evolution of probability density function and direct probability integration, have wide application range, but also high computational cost. (4) The active learning algorithm based on the proxy model, which combines the proxy model with stochastic simulation method through active learning, has the advantages of high efficiency of the proxy model and high accuracy of the stochastic simulation method, and is one of the research hotspots in the world in recent years.

In recent years, scholars at home and abroad have studied the reliability evaluation of grain. Ren studied the analysis of structural reliability calculation method of SRM [122]. Liu and Wang assumed that the material properties and loads were random, and analyzed the structural reliability of the nozzle expansion section of SRM [123]. Because of the viscoelasticity of propellant materials, this paper summarizes the research situation of uncertain structure analysis of some viscoelastic materials. Hilton et al. extended the elastic-viscoelastic correspondence principle to random processes caused by random linear viscoelastic materials, discussed the correspondence principle of separated variables and integral transformation, and studied the randomness of creep and relaxation functions by using Gaussian distribution and Beta distribution [124]. Burczyn et al. [125] applied the correspondence principle and boundary element method to study the random dynamic response of viscoelastic problems. Gutie’rrez and De Borst use elastic-viscoelasticity model and finite element reliability method to analyze the influence of random material defects on localization behavior [126]. Ditlevsen proposed a constitutive equation of random white noise type, which is consistent with the commonly used linear viscoelastic theory in the mean sense [127]. Heller et al. [128] describe the storage environment temperature of grain as Gaussian narrow wave random process, give the stress–strain solution of hollow cylindrical elastic grain in analytical form, analyze the statistical properties of strength, stress and strain, and give the failure probability of grain. Humble also estimated the failure probability of SRM grain under random temperature environment [129]. Lee et al. [130] proposed a simplified stochastic temperature model to estimate the temperature damage storage reliability of solid rocket propellant. Li et al. [131] took the solidification cooling of SRM grain as an example and studied the reliability of failure correlation between inner surface crack and insulation layer debonding. In order to improve the calculation accuracy and reduce the calculation cost, a failure-related reliability analysis method based on PCE is proposed. Yålmaz et al. [132] considered the uncertainties of load and material parameters, and the linear viscoelastic material model was adopted for the propellant. Laheru cumulative damage model is used to represent the degradation of structural performance, and Layton model is used to simulate the aging effect. The alternative mathematical models of induced stress and strain are established by response surface method, and the limit state function is established to predict failure. The instantaneous reliability is calculated within a certain confidence interval by first-order reliability method. Gang et al. [133] analyzed the reliability of bolt connection and O-ring seal of SRM and estimated the input variables statistically by Bayesian method. The estimated variables and failure probability were calculated by reliability analysis. Raouf et al. [134] modeled the SRM as a shell with uniform thickness composed of ellipsoidal head and cylindrical body subjected to ICP, which indicated that the cylindrical part was stressed from circumferential and longitudinal directions, thus carrying out reliability analysis. Moon uses FMECA method to quantitatively determine the reliability of components, and integrates it into fault tree analysis, so as to obtain the reliability of the system. The quantitative FMECA is realized by burden method and ability method. Otherwise, use the failure rate manual for semi-quantitative FMECA. The four most important problems of SRM are selected to explain the load and capacity method, namely, fracture caused by failure of connecting bolt and O-ring seal, shell fracture and leakage, and the reliability prediction method of SRM based on probability system is studied [135]. Tan et al. [136] considered the randomness of some parameters, and listed the simulation program block diagram of grain reliability based on axisymmetric linear elastic finite element theory, which is actually the application of response surface method, in which the viscoelasticity of materials is not considered, and the theoretical formula derivation and example calculation are not carried out. Liu studied the reliability of cumulative damage of grain strength under random load [137]. Based on the treatment of propellant elongation principal curve, Wang analyzed the structural integrity and reliability of grain by empirical formula [138]. Zhang used two-dimensional viscoelastic stochastic finite element based on approximate incompressibility to analyze the reliability of grain [139]. Zhu used the linear cumulative damage model proposed by Bills and Wiegand to calculate the failure probability of grain structure under temperature shock and temperature cycle [140]. Zhang et al. [141] also carried out similar digital simulation of solid propellant grain strength reliability by Monte Carlo method. Xu et al. [142] analyzed the random vibration response of solid motor grain in road transportation environment but did not calculate the fatigue cumulative damage in long-term road transportation environment. Aiming at the randomness and dispersion of mechanical properties of propellant materials, Wang constructed a stochastic constitutive model and carried out the structural integrity evaluation of grain based on the stochastic constitutive model; In order to evaluate the reliability of grain structure with both random variables and interval variables, a probabilistic-non-probabilistic hybrid reliability model is adopted to calculate the reliability of grain under ignition and pressurization conditions [143]. Chen et al. [144] analyzed the structural reliability of solid propellant grain on the basis of safety factor method, combined with stress intensity interference theory and response surface method. The results obtained by this method comprehensively considered the randomness of variables, avoided greater subjective randomness, and had great application value in the reliability analysis of solid propellant grain.

To sum up, reliability technologies based on statistical data and failure physics have been widely developed at present. Both types of technologies have their own advantages and disadvantages. The statistical method based on failure data is difficult to accurately obtain the distribution characteristics of product life because of the small number of sub-samples, and the difficulty of the reliability evaluation method based on failure physics is that it is difficult to accurately simulate the physical process of performance degradation and failure. Therefore, reliability prediction technology combining failure data with failure physics has gradually become a research hotspot in recent years. It is a feasible way to realize highly credible reliability evaluation by collecting physical quantities related to failure mechanisms during the reliability test, revising the failure physical model, and fusing failure data with reliability prediction information of the physical model. The key technology lies in establishing high-precision failure physical model and unifying multi-source data of parameter uncertainty model.

## 4. Challenges Faced by Reliability Analysis of SRM Grain

### 4.1. Problems in Reliability Research of SRM Grain

At present, there is still no effective reliability design system for solid motor grain in China. In recent years, with the accumulation and development of technology, the independent design ability has been greatly improved, but the positive design system for product research and development is still not perfect. At present, the industry pays little attention to the guidance of reliability index in the whole product design process, only pays attention to the product performance, and ignores the important influence of life and reliability on the product development process [2].

At present, the shortage of reliability design system of solid motor grain in industry is that reliability is not considered in every design link, and its reliability index (mainly life) is preliminarily verified only when the product is tested and accepted, ignoring the core idea that reliability should run through the whole design process of the product [145]. In addition, at present, an effective and comprehensive reliability database of solid motor grain has not been established in the whole industry, whether it is for the same product or similar products. There are many subjective and objective uncertainties in the remaining data, such as unclear data, incomplete data, wrong records and so on, which can not effectively use abundant resources in the design, and has not formed an effective iteration of reliability design. In addition, the collaborative design of performance, life and reliability parameters is not involved, and the design of each stage does not implement the standard and feasible reliability design criteria [146].

Reliability engineering technology has become a research hotspot in the international mechanical field. The United States has invested huge manpower and financial resources in missile reliability research, carried out a large number of fault mechanism research, design technology research and test technology research, and deeply explored the key factors affecting system reliability. For example, in view of the failure caused by the performance degradation of Trident submarine-launched missile parts, the United States implemented the life extension improvement technology, redesigned the guidance system, upgraded and replaced the components, and incorporated new technologies to further enhance the technical performance, combat capability and reliability of the missile weapon itself. For the grain with high reliability and long life, Western developed countries focus on the comprehensive design technology research of performance and reliability, adopt the measures of combining pre-research with model application, constantly sum up experience, introduce new technology, continuously popularize and apply it in product design, and closely explore related technologies around the aspects of long structural life, high reliability and strong applicability, and obtain mature and fruitful research results, showing that the storage life and reliability of foreign similar products are far ahead of those of domestic similar products [147].

Compared with European and American space power countries, China’s grain reliability technology still has the following problems [6,64,148,149]:

(1) In terms of technical requirements, China’s SRM technology is in the stage of catching up and surpassing to independent innovation. Scientific researchers generally realize the importance of product reliability. In the early stage, the application of reliability was not paid attention to, which led to prominent failure problems. Although the importance of reliability has been fully paid attention to at present, the inertia thinking of early research and development design is difficult to change immediately. At present, the design process of existing models only considers relevant reliability problems locally, and reliability technology has not been integrated into the whole development process of products.
(2) SRM grain can learn from the reliability analysis, sensitivity design and reliability optimization design of mechanical products. However, for mechanical products, there are still some shortcomings in modeling and model modification methods based on typical fault mechanisms such as aging, and key technologies such as simulation calculation of time-varying wear-out fault characteristics, calculation of gradual reliability sensitivity and efficient methods of time-varying reliability analysis have not been completely broken through. Compared with the technical level of similar products abroad, the theoretical research foundation of solid motor grain reliability in China is relatively weak, and the application and effectiveness of new technologies still need to be further strengthened.
(3) In engineering applications, the application of reliability technology in solid motor product research and development is still not comprehensive, and most of them are local applications of reliability-related technologies, such as fault diagnosis technology, fault tree analysis, reliability test appraisal and other related application research. At present, there is no application from the whole life cycle of product research and development, production, storage and use. In terms of reliability standards, foreign standards are tracked and used for reference, and few standards are independently formulated based on independent design experience and innovative technology.
(4) There is a gap between theory and engineering application because there are still many problems to be solved in mechanical reliability and many failure mechanisms are unclear in the process of mechanical reliability research. Although there is endless theoretical research on reliability, the correlation degree with actual products is weak, and there is a lack of engineering applications, especially demonstration applications. The essence is that the failure mechanism of products and their parts is not proven, the reliability failure data of mechanical products is not systematically established, and the limit state equation for reliability design evaluation and analysis is difficult to accurately establish. These factors are also the most important problems that restrict the reliability development of solid motor grain in China for a long time.

### 4.2. Main Technical Challenges of SRM Grain Reliability Research

At present, the performance and reliability design analysis, reliability optimization theory, reliability test method and evaluation method in the research and development of solid motor grain have not been comprehensively applied. SRM is developing in the direction of long life and high reliability, and the integrated design of grain performance and reliability should be carried out, which mainly faces the following technical difficulties and challenges [19,150,151]:

(1) The study of randomness of load spectrum of grain under working conditions is insufficient.

Grain usually bears variable stress load spectrum. In order to ensure the accuracy of life model modeling, it is necessary to give its conventional load spectrum statistics and conversion method based on working conditions. Because the actual working conditions are usually staged loads, there are certain impact loads in the process of different load switching, and the loads borne by grain under different working conditions have certain randomness. Therefore, in the conventional load spectrum statistics and conversion process, it is necessary to fully consider the load stages, the impact in the load switching process and the randomness of the load of different grain under different working conditions, and consider the influence of the load action sequence on the cumulative damage effect.

(2) The failure mechanism of grain time-varying wear failure is unclear.

The failure of the grain usually occurs after the engine has been stored for quite a long time, and these failures are often related to the performance loss, degradation and aging of the grain. At present, some simulation and experimental studies have been carried out on several main wear-out fault mechanisms in China. The time-varying wear fault characteristics of mechanical parts are simulated by simulation solution technology. At the same time, in the time history of the product, the output performance such as stress and strain can be predicted from the simulation. However, due to the limitation of test conditions, simulation technology has not been effectively verified by experiments, especially in the simulation technology of high-precision characteristics such as performance degradation and aging, which leads to the low maturity of simulation technology and cannot directly guide engineering design.

(3) The integrated design system of grain performance and reliability has not been established yet.

With the development of solid motor technology, the reliability of grain performance is required to be higher and higher. On the basis of high performance, in order to achieve the design goal of high reliability and long life, it is necessary to study the optimization strategy of performance and reliability at the same time. At present, the optimization design of solid motor grain is mainly based on local optimization, and the overall optimization research of grain is not systematically carried out. Therefore, how to achieve the optimization goal of long life and high reliability on the basis of high performance is an important challenge in the research of grain reliability.

(4) It is difficult to accurately evaluate the reliability of solid motor grain under very small samples.

There are some problems in the evaluation of grain reliability parameters, such as small samples and large uncertainty, which lead to great difficulty in its reliability evaluation. The Bayes method can use probability density function to describe the uncertainty degree of parameters and can update the certainty degree of the established model continuously according to the mastered information, thus improving the accuracy of reliability evaluation results of small sample products. However, in the actual process, the prior distribution of grain reliability parameters is generally based on the existing information (prior information) to choose the appropriate distribution model fitting and cannot guarantee the conjugation of prior distribution and overall distribution. In the Bayes evaluation process of non-conjugate prior distribution, the key problem is to determine the posterior distribution of reliability parameters. When there are many unknown parameters in the posterior distribution, it is very difficult to filter out the redundant parameters by integral and then obtain the edge posterior distribution of the parameters to be estimated.

(5) The reliability of accelerated life model of grain has not been fully verified.

The accelerated life test technology of grain is relatively weak, and the main methods refer to the related technologies of foreign accelerated life test, but the selection of acceleration factor, acceleration model, the number of accelerated test pieces and the determination of acceleration ratio in accelerated life test have not been effectively verified, and an effective and feasible accelerated life test specification has not been established.

### 4.3. Research and Development Direction of SRM Grain Reliability

As the key structure of solid motor, the performance design is considered in the traditional design, and the reliability design and evaluation are not integrated into the product design, which leads to the low life and reliability index of the current solid motor grain and can not meet the high reliability working requirements of solid motor. As far as reliability theory research is concerned, the related theoretical research and simulation have been relatively mature, even leading the theoretical methods to solve the actual product needs. For a long time, the reliability of mechanical products has been difficult to integrate into the process of product development and design. On the one hand, the physical failure mechanism in the actual operation process of products has not been fully proved; On the other hand, it is difficult to obtain the test data of mechanical product reliability research or accumulate relevant test data. The cost is too expensive, and the cycle is too long, which leads to difficulty in applying reliability design theory to engineering practice.

In order to better apply the reliability theory to the R & D system of products, the key points of solid motor grain design in China, as well as the problems, challenges and suggestions are given with reference to other advanced reliability design processes. In the stage of product development and design, from the point of view of system engineering, reliability design methods, analysis methods, simulation methods and test methods should be implemented. Specialized flow must be integrated into the design system in every stage of scheme demonstration, preliminary design and detailed design, including the statistics and analysis of reliability history information, the implementation and implementation of engineering technologies such as reliability allocation and prediction, the simulation calculation of wear-type fault characteristics that seriously affect life, the simulation analysis of life and reliability, the test verification and evaluation of product life and reliability, and the specific measures to improve reliability such as FMECA, GO method and FTA in the whole design process.

Based on the above analysis, in order to better apply the reliability theory and method to the research and development of solid motor grain, the following research work needs to be strengthened [131,152,153]:

(1) At the initial stage of product research and development, attention should be paid to the collaborative design and evaluation of grain performance and reliability. On the basis of proving the failure mechanism of grain, the simulation calculation model of failure mechanism should be studied, and the experimental verification of simulation failure mechanism model should be strengthened. Combined with the technical maturity requirements of different stages, experimental verification technology should be deepened step by step to accumulate a large number of experimental data. Only the simulation technology verified by experiments can really guide product design, so as to find out the failure evolution process of grain from two dimensions of physical mechanism and test data and lay a foundation for subsequent reliability design analysis.
(2) Attention should be paid to solving two kinds of problems: deterministic design and uncertain design. After completing and realizing deterministic design such as product performance and life in the process of finding out failure mechanism, the research on uncertain design of grain should also be carried out. Uncertainty usually includes subjective uncertainty and objective uncertainty. In the research process, it is necessary to carry out probability representation by correlation probability method, and complete reliability analysis and design based on proxy model.
(3) Attach importance to the integrated design and evaluation of product performance and reliability. Establish an integrated optimization design framework considering the performance, life and reliability of grain, and complete the overall collaborative framework design with performance meeting requirements, long life and high reliability. A reliability evaluation model based on prior information and test data is established to evaluate the reliability parameters of grain.
(4) In terms of the theoretical methods for advancing the reliability research of propellant grains, considering the characteristics of small-batch and high-value products with grain structures, efficient small-sample reliability analysis methods should be studied. For the robust design of grain performance, robust design research can be conducted. In addition, by combining machine learning methods, efficient reliability analysis methods based on machine learning can be studied. By applying new artificial intelligence technologies, these methods can be applied to the reliability research of grain structures, thereby expanding the forward design system of grains.

Product research and development is a systematic project, involving basic theory and engineering technology, covering materials, processes, simulation, tests and other links. The above research suggestions are only put forward from the perspective of reliability research, and the relevant research suggestions cannot take into account the research work of all disciplines. The implementation and application of the final theory and method should be guided by the actual needs of enterprises and jointly carried out by relevant researchers, so as to form a reliability design criterion that can guide the industry to establish grain, and lay a technical foundation for the forward design and engineering development of high-reliability grain.

## 5. Conclusions

At present, the life and reliability of grain are obviously uncertainty. As the development trend of modern advanced aerospace power technology, the comprehensive design technology of grain performance and reliability is one of the key technologies that must be broken through in the field of aerospace power in China. At present, foreign advanced power countries have adopted the measures of combining pre-research with model application to study the comprehensive design technology of grain with high reliability, long life and low cost, constantly summing up experience, introducing new technologies, and constantly popularizing and applying them in product design. In the field of solid motor in China, there is still a gap between the technical level and that of western developed countries: the failure mechanism of grain products has not been fully proved, the basic database for product reliability research is not sufficient, and the basic, universal and strategic position and role of product reliability engineering technology are not paid enough attention, resulting in a significant gap between the development of related technologies in China and foreign countries. In order to shorten the gap with foreign countries and accelerate the realization of independent, advanced technology and reliable quality solid motor grain, research work should be carried out on the performance and reliability collaborative design of high-reliability and long-life grain based on forward design thinking. On the basis of truly finding out the failure mechanism of various faults of products, design criteria, optimization methods and test databases of product performance and reliability should be formed, which will lay a technical foundation for the forward design and engineering development of SRM grain.

## Figures and Tables

**Table 1:** Influencing factors of grain performance degradation caused by SRM aging.

Influencing Factors		Form of Expression	Failure Mode
Chemical state change	1. Chemical reactions occur between a single component of the propellant or a combination of propellants.	Hardening, catalysis, venting, accumulation of degradation products, enhanced viscous flow, and changes in adhesion.	The possibility of cracks occurring during storage, ignition or temperature cycling increases: the burning rate may change; Specific impact loss Ignition issues and liner debonding.
	2. Undergo chemical reactions with the environment: (1) Atmosphere: Humidity. Gaseous or solid decomposition products (autocatalytic reaction). Air, oxygen, ozone, and pollutants in the air. (2) Other materials in the engine.	The same as (1): In addition, the inhomogeneity that the propellant has on the surface and in the body.	The same as (1).
	3. Factors that may affect the rate of change: (1) Temperature. (2) Stress state.	The same as (1).	The same as (1).
	4. Radiation	-	-
	5. Bacterial action	Surface changes in polymer cross-linking or degradation.	The same as (1).
Change in physical state	1. Reversible physical changes: (1) Phase change is related to time and temperature. (2) Elastic strain. (3) Material diffusion: Gas. Plasticizer. Humidity.	The physical properties related to temperature undergo hysteresis; it might be very small; the performance of the propellant is uneven, porous and shrinking.	The possibility of cracks occurring during storage, ignition or temperature cycling increases; it might be very small; crack growth increases the possibility of cracks appearing during storage, ignition, or temperature cycling.
	2. Irreversible physical changes. The strain caused by the following factors exceeds the reversible range: a. Gravity. b. Acceleration (Transportation). c. Thermal gradient ambient temperature.	Crack, lining debonding, viscous deformation, dehumidification (bleaching).	The burning surface and burning rate increase.

## Data Availability

Data for replication can be provided upon request.

## Abbreviations

The following abbreviations are used in this paper:

SRM	Solid Rocket Motor
HTPB	Hydroxy-Terminated Liquid Polybutadiene Rubber
FMECA	Failure Mode, Effects and Criticality Analysis
FTA	Fault Tree Analysis
PCE	Polynomial Chaos Expansion
Nomenclature	
*T*_0_	Initial temperature of 0 degrees stress temperature
*ε_θ_*	Maximum circumferential strain
*ε_m_*	Maximum stretching elongation
*γ_8_*	Octahedral she ar strain
*γ_8m_*	Octahedral shear strain critical value
*ε_v_*	Von Mises strain
*σ_n_*	Normal tensile stress
*τ_t_*	Tangential shear stress
*u_i_*	Deformation
*K_ig_*	Stress intensity factor
*J*_g_	*J* integra
*G_g_*	Energy release rate
*K_igC_*,	Fracture toughness
*J_gIC_*	Critical J
*R_gIC_*	Propagation resistance of grain crack
